# MicroRNA let‐7a regulates angiogenesis by targeting *TGFBR3* mRNA

**DOI:** 10.1111/jcmm.13960

**Published:** 2018-11-22

**Authors:** Shao Wang, Huandong Zhou, Dazhou Wu, Huajing Ni, Zhongliang Chen, Chengshui Chen, Youqun Xiang, Kezhi Dai, Xiaoming Chen, Xi Li

**Affiliations:** ^1^ School of Mental Health Wenzhou Medical University Wenzhou China; ^2^ The Affiliated Kangning Hospital of Wenzhou Medical University Wenzhou China; ^3^ The First Affiliated Hospital of Wenzhou Medical University Wenzhou China

**Keywords:** angiogenesis, Let‐7a, migration, TGFBR3, TGFβ signalling

## Abstract

Angiogenesis has a great impact on human health, owing to its participation in development, wound healing and the pathogenesis of several diseases. It has been reported that let‐7a is a tumour suppressor, but whether it plays a role in angiogenesis is unclear. Here we showed that let‐7a, a microRNA conserved in vertebrates, regulated angiogenesis by concomitantly down‐regulating TGFBR3. Overexpression of let‐7a or knockdown of TGFBR3 in cell culture inhibited the tube formation and reduced migration rate. Moreover, xenograft experiments showed that overexpression of let‐7a or knockdown of TGFBR3 had smaller tumour size. Downstream genes, such as VEGFC and MMP9, were also down‐regulated in let‐7a overexpression or TGFBR3 knockdown groups. Therefore, our results revealed a novel mechanism that let‐7a regulate angiogenesis through post‐transcriptional regulation of TGFBR3.

## 1 INTRODUCTION

Angiogenesis is a physiological process in which new blood vessels form from existing vessels. It is generally deduced that angiogenesis is regulated by a balance between pro‐ and anti‐angiogenic cues.^1^, ^2^ When dysregulated, it has a great influence on pathogenesis of many diseases, such as cancer, inflammation, infectious disease and congenital or inherited diseases.^3^, ^4^

Transforming growth factor‐beta (TGFβ) superfamily members have three different types of receptors, but exhibit particularly high affinity to type II transmembrane serine/threonine kinase receptors (TGFBR2).^5^ Upon ligand binding, TGFBR2 recruits and transphosphorylates type I receptors (TGFBR1), which subsequently activate downstream signal mediators, SMADs. Type III receptors (TGFBR3 or ENDOGLIN) enhance ligand binding to their cognate receptors, namely TGFBR1 and TGFBR2, and therefore activate signalling transduction through phosphorylation of SMAD proteins.^6^, ^7^, ^8^ To our interest, Tgfbr3‐null mice died at embryonic day 14.5 due to defective coronary vasculogenesis, of which few evident vessels and persistent blood islands were found throughout the epicardium.^9^ This finding suggests that TGFBR3 may play an important role in regulation of angiogenesis.

MicroRNAs (miRNAs) are a class of small non‐coding RNAs, ~22 nucleotides in length, which regulate gene expression by binding to the 3′‐untranslated regions (3′UTR) of target mRNAs and causing subsequent degradation of the target mRNA and/or inhibition of mRNA translation.^10^, ^11^ The lethal‐7 (let‐7) gene family was initially discovered as an essential developmental module in *Caenorhabditis elegans* and later determined to act as miRNAs in other species.^12^ Follow‐up studies have comprehensively described the diverse biological functions of the 12 members of the let‐7 miRNA family, including cell proliferation,^13^ cell differentiation,^14^ tumorigenesis^15^, ^16^ and angiogenesis.^17^ It has been recently shown that let‐7a, along with other miRNAs may favour the “Off” switch of tumour angiogenesis in a mouse model of breast tumour undergoing hormone therapy.^18^ Intriguingly, TGFBR1, a member of TGFβ family, identified as potential target of let‐7, was critical for endothelium inflammation and cell differentiation.^19^, ^20^, ^21^, ^22^, ^23^

In this study, we focused on identification of let‐7a‐regulated genes involved in angiogenesis, particularly the TGFβs signalling pathway,^24^ and found *TGFBR3* to be a novel target for let‐7a. Also, let‐7a may impair angiogenesis via post‐transcriptional regulation of *TGFBR3*. Our data likely identify a mechanistic explanation for the anti‐angiogenesis function of let‐7a and provide a potential therapeutic target for anti‐ or pro‐angiogenesis strategies in cancers and other diseases.

## 2 MATERIALS AND METHODS

### 2.1 Animals, cell culture and transfection

Nude mice (7‐8 weeks of age) were purchased from the Laboratory Animal Center of Wenzhou Medical University. All outlined in vivo procedures were approved by the Institutional Animal Care and Use Committee of Wenzhou Medical University. The human embryonic kidney 293T (HEK293T) and pancreatic islet endothelial (MS1) cell lines were cultured in Dulbecco modified Eagle medium (DMEM) (Gibco, Waltham, MA, USA) supplemented with 10% foetal bovine serum (FBS) and 1% penicillin/streptomycin at 37°C in a 5% CO_2_ incubation chamber. Human umbilical vein endothelial cells (HUVECs) were cultured in endothelial cell medium (Sciencell, San Diego, CA, USA) supplemented with 5% FBS, 1% endothelial cell growth supplement, 1% penicillin/streptomycin at 37°C in a 5% CO_2_ incubation chamber. When applicable, HEK293T cells and HUVECs were transfected with plasmid DNA, let‐7a negative control (Con mimics) and let‐7a mimics (RiboBio, Guangzhou, China) by using Lipofectamine 2000 or Lipofectamine LTX and Plus Reagent (Invitrogen, Waltham, MA, USA) according to the manufacturer's instructions.

### 2.2 Plasmid construction

To construct the luciferase reporter vectors, a wild‐type 3′UTR (3′UTR‐WT) fragment of human and mouse TGFBR3 containing putative binding sites for let‐7a was amplified from genomic DNA (amplification primers are listed in Table 1). The amplified fragment (1229 bp and 349 bp in length respectively) was first inserted into pGEMT vector for site‐directed mutagenesis. The mutant 3′UTR (3′UTR‐MUT) of TGFBR3, carrying a mutated sequence in the seeding region of let‐7a, was mutated (Primers are listed in Table 1) with the Quick Change Site‐Directed Mutagenesis Kit (Agilent Technologies, Santa Clara, CA, USA). Then, WT and mutated 3′UTR fragments of TGFBR3 were cloned into the pmirGLO vector (Promega, Madison, WI, USA) within PmeI and SalI sites. Both insertions were verified by sequencing (Genscript, Nanjing, China).

**Table 1:** Real‐time PCR primer sequences and 3'UTR fragment primer sequences

Primers	Sequences 5′‐3′
GAPDH	F: TGGGTGGCAGTGATGGCA R: GGAGAAGGCTGGGGCTCAT
TGFBR3	F: AAAGCAGCAGAAGGGTGTGT R: ACCTGGAAAGCACTGTAGGG
VEGFC	F: TGCCGATGCATGTCTAAACT R: TGAACAGGTCTCTTCATCCAGC
MMP9	F: GATGCGTGGAGAGTCGAAAT R: CACCAAACTGGATGACGATG
ID1	F: CACCCTCAACGGCGAGATC R: CCACAGAGCACGTAATTCCTC
PROX1	F: ACAAAAATGGTGGCACGGA R: CCTGATGTACTTCGGAGCCTG
hTGFBR3 3′UTR	F: AGCTTTGTTTAAACAGAAGGGTATCAGAGTGGAGG R: GCTCTAGATCACATAGGACTCACCCAACA
hTGFBR3 3′UTR Mutant primers	F: CTTTTTGTACTGTAACTGCGGCATGGTTTGAATGATG R: CATCATTCAAACCATGCCGCAGTTACAGTACAAAAAG
mTGFBR3 3′UTR	F: AGCTTTGTTTAAACCAGGACTGTCTGTGCAAGGCAC R: GCTCTAGA ATCTGTCAGTTTAATGAACGA
mTGFBR3 3′UTR Mutant primers	F: CTTTTTGTACTGTAATCGTTCTATGGTTTGAATGATG R: CATCATTCAAACCATAGAACGATTACAGTACAAAAAG

### 2.3 Luciferase reporter assay

For the luciferase reporter gene assay, HEK293T cells plated in a 24‐well plate were co‐transfected with 50 nmol/L of either Con mimics or let‐7a mimics with 500 ng reporter comprising 3′UTR‐WT or 3′UTR‐MUT. Two days after the transfection, cells were lysed in Passive Lysis Buffer (Promega) and Firefly and Renilla luciferase activity was measured with a GloMax^®^ 20/20 Luminometer (Promega) using the Dual‐Luciferase^®^ Reporter Assay Kit (Promega) according to the manufacturer's protocols.

### 2.4 RNA isolation and reverse transcription

Total RNAs were isolated from HEK293T cells or HUVECs using TRIZol (Invitrogen) following the manufacturer's instructions. RNA concentration was determined by UV absorbance at 260 nm. Approximately, 1 μg of total RNAs was used for reverse transcription using RevertAid First Strand cDNA Synthesis Kit (Thermo Fisher Scientific, Waltham, MA, USA) according to the manufacturer's instructions.

### 2.5 Quantitation of mRNA expression

Real‐time PCR was carried out in an ABI 7500 system with PowerUp™ SYBR^®^ Green master mix (Thermo Fisher Scientific) following the manufacturer's instructions. Melting curves for each PCR were carefully monitored to avoid non‐specific amplification. Each sample was analysed in triplicate. The expression level of mRNA was normalized with GAPDH expression and relative expression of target genes was calculated using the 2^−ΔΔCt^ method. All primers are listed in Table 1.

### 2.6 Protein extraction and Western blotting analysis

HEK293T, HUVECs and MS1 cells were harvested and lysed in RIPA buffer supplemented with protease inhibitors and phosphatase inhibitors (Beyotime, Shanghai, China). Total protein concentration was quantified by using the BCA Protein Assay Kit (Beyotime). Equal amounts of protein samples (~30 μg) were separated on a 10% SDS‐PAGE and transferred onto a PVDF membrane (Millipore, St Louis, MO, USA). After blocking with 5% non‐fat milk in TBSTween‐20 (TBST) for 2 hours at room temperature, the membranes were incubated with appropriate primary antibodies at a 1:1000 dilution overnight at 4°C. The membranes were washed three times and corresponding horseradish peroxidase‐conjugated secondary antibody diluted at a 1:5000 was added and incubated for 2 hours at room temperature. After another triplicate wash with TBST, the PVDF membranes were developed with an enhanced chemiluminescent assay Kit (Thermo Fisher Scientific) according to the manufacturer's protocol. Resulting protein bands on the membranes were visualized with a ChemiDoc™ XRS+ imaging system (Bio‐Rad, Hercules, CA, USA). The intensity of the bands was quantified by using Image J (Media Cybernetics Inc., Rockville, MD, USA). Anti‐Vinculin (Sigma, St Louis, MO, USA) and anti‐TGFBR3 (Abcam, Cambridge, MA, USA) primary antibodies were used.

### 2.7 Tube formation assay

The HUVECs tube formation assay was performed with Matrigel (BD Bioscience, Franklin Lakes, NJ, USA) according to the manufacturer's instructions. The sterile 96‐well plates were coated with 50 μL Matrigel and incubated at 37°C for 1 hour to form gels. Let‐7a mimics‐transfected (or siRNA against TGFBR3) and control HUVECs (1 × 10^5^) were seeded into each well and incubated with cell‐free culture supernatants from HUVECs at 37°C in a 5% CO_2_ incubator for 6 hours. The degree of tube formation was evaluated using an inverted microscope (Olympus, Tokyo, Japan) and the number of tubes was calculated using Image J software (Media Cybernetics Inc.).

### 2.8 Wound‐healing assay

HUVECs were treated with miRNA/siRNA as described above. Wounds were introduced by scrapping with a pipette tip after a 48 hours incubation when a monolayer culture was formed, and microscope photography captured images immediately (0 hour) and 6 hours after. The wound gaps at both time points were measured to be represented as a migration index, for example the distance migrated by let‐7a mimics/TGFBR3 siRNA‐treated cells relative to the distance migrated by NC‐treated cells.^25^

### 2.9 Murine tumorigenesis

To generate hemangioendothelioma, the injection of MS1 cells in mice was performed as previously described with minor modifications.^26^ Briefly, MS1 cells were collected, resuspended in DMEM medium (4.5 × 10^5^ cells in 200 μL) and subjected to dorsally subcutaneous injection in nude mice. For knockdown of Tgfbr3 or overexpression of let‐7a, MS1 cells were first transfected with Tgfbr3 siRNA or let‐7a mimics for 48 hours prior to injection. Mice were killed 2 weeks post injection. Following skin detachment, hemangioendothelioma tissues were harvested and the extra fluid was removed to assess tumour weights.

### 2.10 Statistical analysis

All experiments were independently repeated at least three times. Quantitative RT‐PCR (qRT‐PCR), luciferase reporter assays and tube formation assays were performed in triplicate. All data were presented as mean ± SEM. The statistical analysis was performed using IBM SPSS Statistics 20 (IBM, Armonk, NY, USA) and GraphPad Prism 6 (Graphpad, La Jolla, CA, USA). The differences were considered statistically significant at *P* < 0.05 based on Student's *t* tests.

## 3 RESULTS

### 3.1 Identification of *TGFBR3* as a novel target of let‐7a

**Figure 1:**
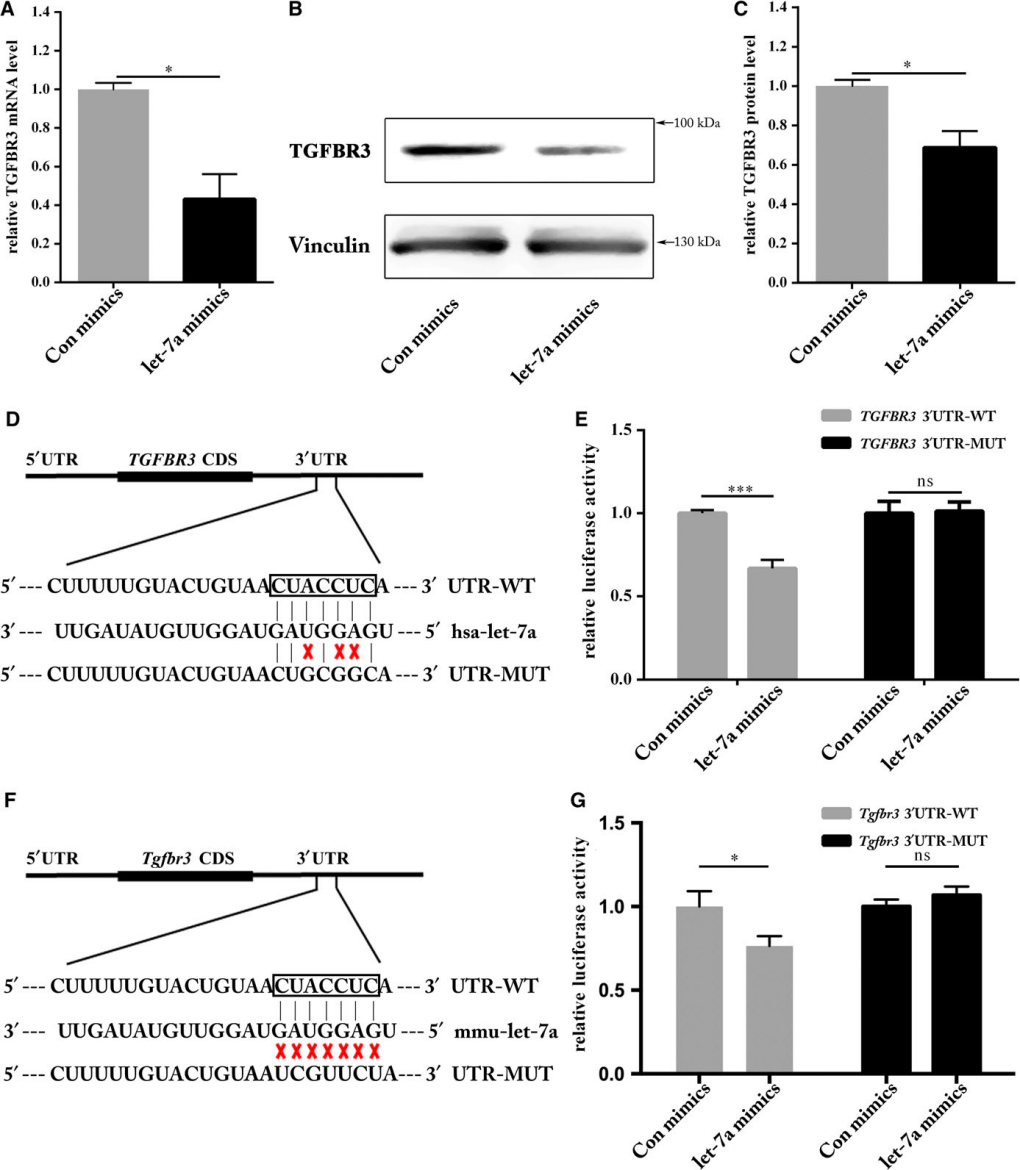
Identification of TGFBR3 as a novel target of let‐7a. A, qRT‐PCR analysis of the TGFBR3 mRNA level was significantly reduced (*P < 0.05). B, Western blot analysis showed reduction in TGFBR3 protein. C, The results of Western blotting were calculated using Image J and Vinculin was used as an internal control. Negative control expression was set as 100% (*P < 0.05). D, A schematic of the computational predicted seed region in the 3′UTR of TGFBR3 was shown, as well as the mutated sequences used in this study. E, HEK293T cells were co‐transfected with either let‐7a mimics or Con mimics and pmirGLO Vector comprising TGFBR3 3′UTR‐WT or ‐MUT. The relative firefly luciferase activity normalized with Renilla luciferase was measured 48 h after transfection (***P < 0.001, ns means no significance). F, Schematic of let‐7a potential binding site in Tgfbr3 3′UTR region. G, The relative luciferase activity mediated by let‐7a and TGFBR3 3′UTR/mutated 3′UTR (*P < 0.05, ns means no significance)

We chose a multitude of candidate genes with high miRNA:mRNA alignment scores predicted by TargetScan and miRanda Web Servers. *TGFBR3* emerged as a potential target gene of let‐7a after screening candidate gene expression with qRT‐PCR of let‐7a mimics‐transfected HEK293T cells. We observed approximately 60% reduction in *TGFBR3* mRNA levels after overexpression of let‐7a (Figure 1A). Reduced mRNA also resulted in decreased TGFBR3 protein levels (Figure 1B and C).

In order to verify that *TGFBR3* is a direct target gene of let‐7a, we cloned the 3′ untranslated region (3′UTR‐WT) of *TGFBR3* mRNA into the pmirGLO vector and then mutated the putative let‐7a binding site by site‐directed mutagenesis (3′UTR‐MUT), as indicated in Figure 1D. As predicted, overexpression of let‐7a significantly inhibited the luciferase activity mediated by 3′UTR‐WT, while mutation of the let‐7a binding site abolished this inhibitory effect (Figure 1E), suggesting that *TGFBR3* was indeed a target gene of let‐7a. Targetscan identified that mouse TGFBR3 3′UTR region had a potential binding site for let‐7a (Figure 1F). Compared to control mimics group, let‐7a could restrain *Tgfbr3* WT 3′UTR fused luciferase activity, but not *Tgfbr3* mut 3′UTR (Figure 1G). This result indicates that mouse *Tgfbr3* is a target of let‐7a.

### 3.2 Regulation of cell migration by let‐7a and TGFBR3 in HUVECs

**Figure 2:**
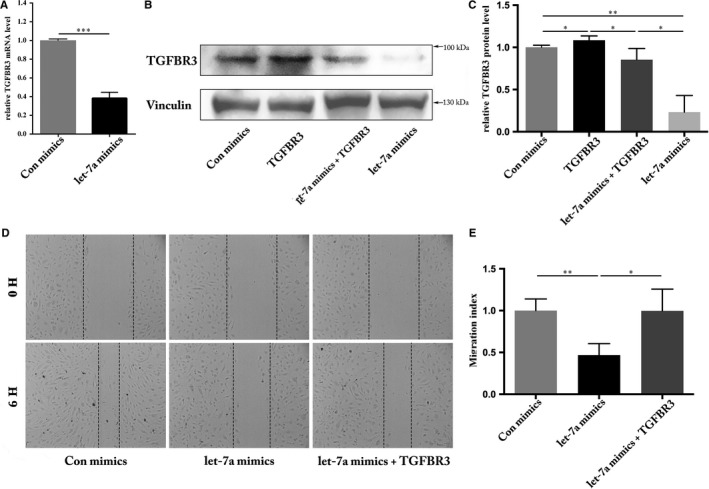
Regulation of cell migration by let‐7a in HUVECs. A, qRT‐PCR analysis of the TGFBR3 mRNA level was significantly reduced (***P < 0.001). B, Western blot analysis showed reduction in TGFBR3 protein. C, The results of Western blotting were calculated using Image J. Vinculin was used as an internal control. Negative control expression was set as 100% (*P < 0.05, **P < 0.01). D, Wound healing assay was performed 48 h after transfection, and the distance between the wound edges was observed and photographed. E, The distance between the wound edges was evaluated using Image J from three independent experiments and is expressed as the percentage of the initial wound distance, n = 3 (**P < 0.01, *P < 0.05)

**Figure 3:**
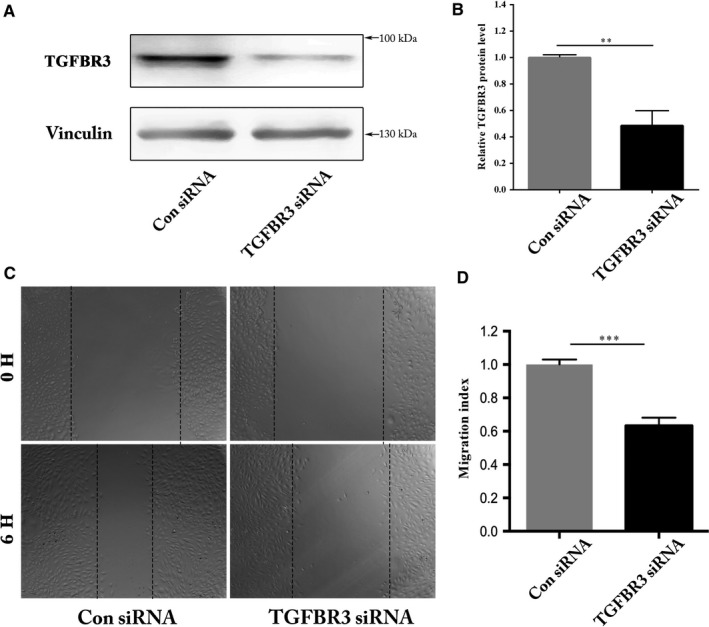
Regulation of cell migration by TGFBR3 in HUVECs. A, Western blot analysis showed reduction in TGFBR3 protein. B, The results of Western blotting were calculated using Image J and Vinculin was used as an internal control. Negative control expression was set as 100% (**P < 0.01). C, Wound healing assay was performed 48 h after transfection, and the distance between the wound edges was observed and photographed. D, The distance between the wound edges was evaluated using Image J from three independent experiments and expressed as the percentage of the initial wound distance, n = 4 (***P < 0.001)

Expression of *TGFBR3* was examined in HUVEC culture using a similar transfection method described above. Concordant with results obtained in 293T cells (Figure 1A‐C), transfection with let‐7a mimics significantly inhibited both TGFBR3 mRNA and protein expression levels (Figure 2A‐C). Studies have implicated that let‐7a mediates cell migration of a variety of cells,^15^, ^27^, ^28^ so we employed a wound‐healing assay to examine migration of let‐7a mimics‐transfected HUVECs. Consistent with previous studies,^15^ migration rates of HUVECs were significantly reduced after the overexpression of let‐7a (Figure 2D and E). As we identified *TGFBR3* as one of the target genes of let‐7a, an obvious question to be answered is whether knockdown of *TGFBR3* has a similar effect on cell migration to that of let‐7a overexpression. *TGFBR3* expression was knocked down with siRNA transfection in HUVECs and the knockdown efficiency was confirmed (Figure 3A and B). As expected, knockdown of *TGFBR3* decreased HUVEC migration (Figure 3C and D), phenocopying defective migration by let‐7a overexpression in these cells (Figure 2C and D). These results indicate that TGFBR3 might be a key mediator of let‐7a‐mediated migration in HUVECs.

### 3.3 Regulation of angiogenesis by let‐7a and TGFBR3 in HUVECs

**Figure 4:**
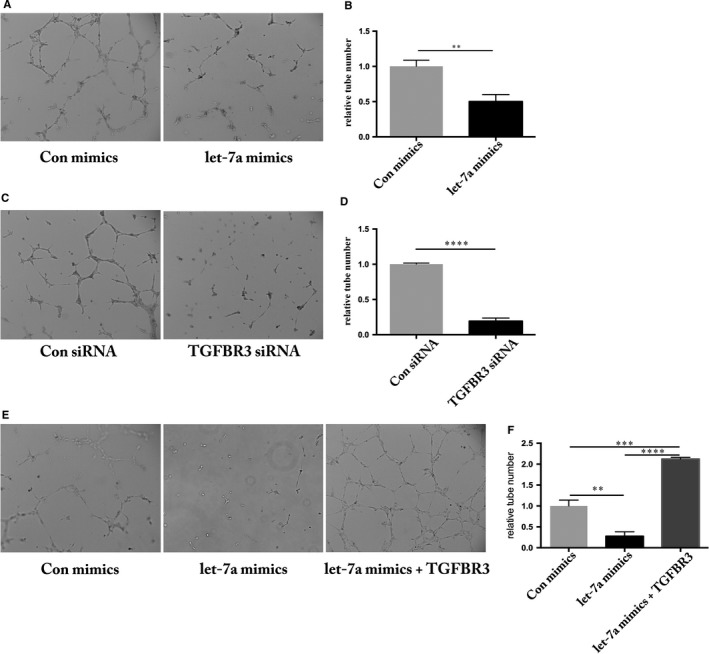
Modulation of angiogenesis by let‐7a and TGFBR3 in HUVECs. A, HUVECs transfected with let‐7a were subjected to tube‐like network formation in matrigel. After 6 h, tube‐like network formation was observed and photographed. B, The tube number was evaluated using Image J and let‐7a mimics group was normalized by the Con mimics (negative control) group, n = 3 (**P < 0.01). C, HUVECs transfected with siRNA were subjected to tube‐like network formation in matrigel. After 6 h, tube‐like network formation was observed and photographed. D, The tube number was evaluated using Image J and TGFBR3 siRNA group was normalized to the Con siRNA group, n = 3 (****P < 0.0001). E, HUVECs transfected with let‐7a mimics and co‐injection with let‐7a and TGFBR3 clone were subjected to tube‐like network formation in matrigel. After 6 h, tube‐like network formation was observed and photographed. F, The tube number was evaluated using Image J, let‐7a mimics group and let‐7a mimics+TGFBR3 clone was normalized to the Con mimics group, n = 3 (**P < 0.01, ***P < 0.001, ****P < 0.0001)

Previous reports have found that let‐7a plays a role in regulating angiogenesis.^17^, ^29^ We carried out a tube formation assay to determine if HUVEC angiogenic ability is affected by let‐7a overexpression. We found that let‐7a mimics the significantly lowered angiogenic activity of HUVECs (Figure 4A and B). siRNA‐mediated knockdown of the let‐7a target gene *TGFBR3* in HUVECs resulted in a similar phenotype to that of overexpression of let‐7a (Figure 4C and D). Furthermore, we found that overexpression of TGFBR3 could rescue the angiogenic activity mediated by let‐7a mimics (Figure 4E and F). Together with data from the wound‐healing assay, we confirmed that let‐7a and TGFBR3 regulated angiogenesis in a linear pathway. Identifying the let‐7a/TGFBR3 axis may explain the anti‐angiogenic function of let‐7a.

### 3.4 Let‐7a/TGFBR3 signalling is required for hemangioendothelioma growth

**Figure 5:**
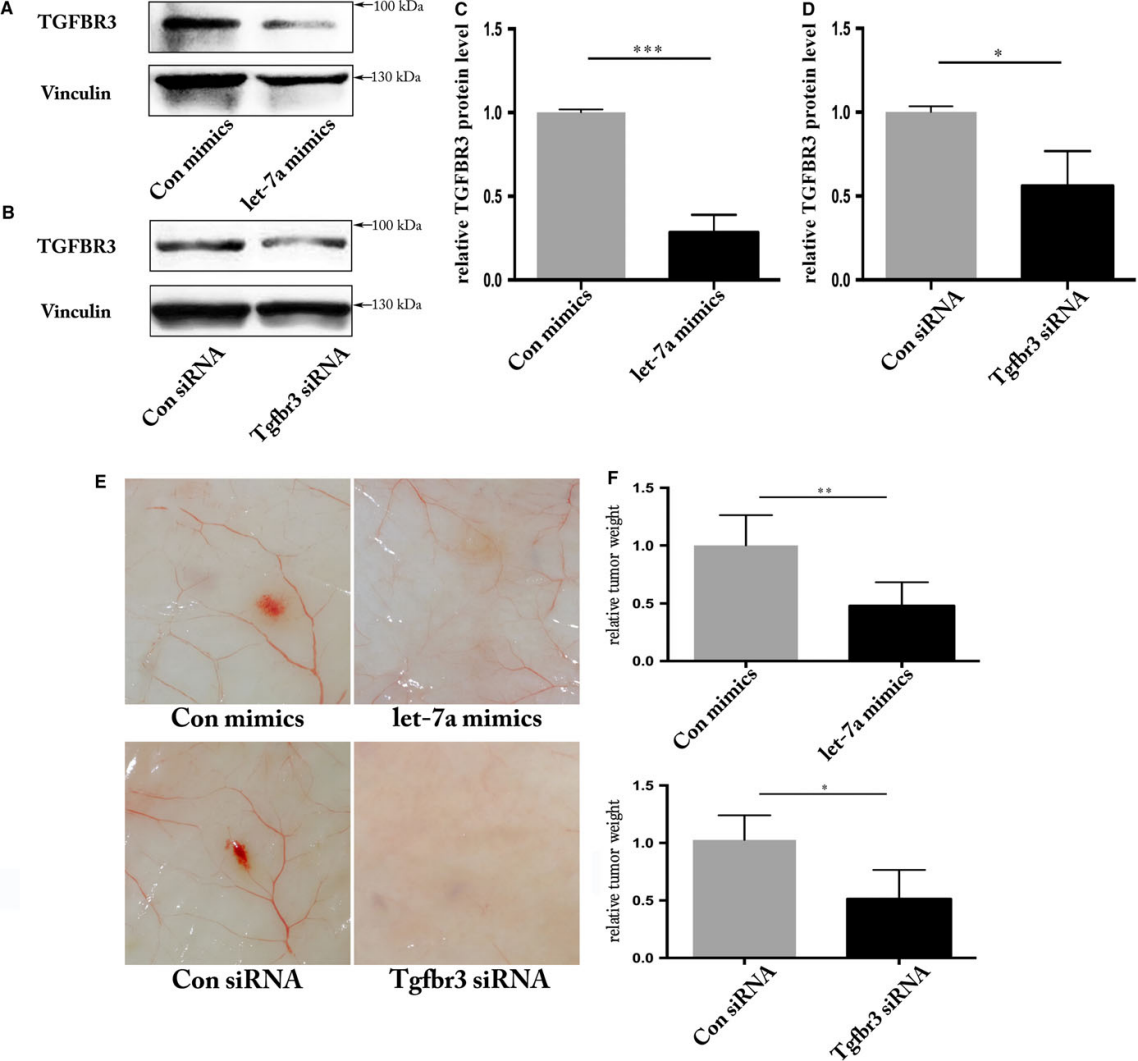
Let‐7a/TGFBR3 is required for hemangioendothelioma growth. Evaluation of TGFBR3 protein level by Western blot after transfection of let‐7a mimics (A) or TGFBR3 siRNA (B) in MS1 cell line. C and D, Quantification of TGFBR3 protein levels from three independent experiments by Image J and Vinculin was used as an internal control. Negative control expression was set as 100% (***P < 0.001, *P < 0.05). E, Representative images showing xenograft hemangioendothelioma in response to the knockdown of TGFBR3 or overexpression of let‐7a (F) the tumour weights in response to the knockdown of TGFBR3 or overexpression of let‐7a, n = 6 (**P < 0.01, *P < 0.05)

To further validate the let‐7a/TGFBR3 axis in vivo, we utilized the MS1 cell line. Overexpression of let‐7a suppressed TGFBR protein levels by ~70% and siRNA‐mediated knockdown of TGFBR3 gave 40% reduction of TGFBR3 protein levels (Figure 5A‐D). Overexpression of let‐7a mimics resulted in smaller hemangioendothelioma, which is similar to knockdown of Tgfbr3 (Figure 5E and F). These results suggest that let‐7a targets Tgfbr3 in vitro and in vivo.

### 3.5 Downstream genes of let‐7a/TGFBR3 axis were dysregulated

**Figure 6:**
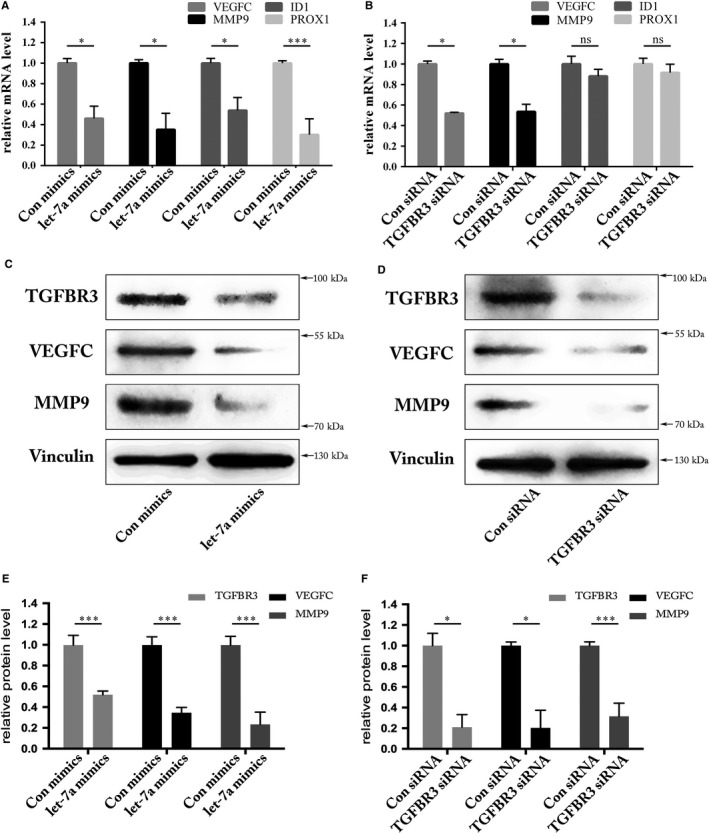
Screening of downstream genes of let‐7a/TGFBR3 axis. A, qRT‐PCR analysis of relative genes showed that mRNA levels were significantly reduced in HUVECs transfected with let‐7a mimics (*P < 0.05, ***P < 0.001). B, qRT‐PCR analysis of the VEGFC and MMP9 mRNA level was significantly reduced in HUVECs transfected with TGFBR3 siRNA, but changes of the ID1 and PROX1 mRNA levels were of no significance (*P < 0.05, ns means no significance). C, Western blot analysis of relative genes showed that VEGFC and MMP9 protein level were significantly reduced in HUVECs transfected with let‐7a mimics. D, Western blot analysis of the VEGFC and MMP9 protein level was significantly reduced in HUVECs transfected with TGFBR3 siRNA. E and F, Quantification of TGFBR3, VEGFC, and MMP9 protein levels from three independent experiments by Image J and Vinculin was used as an internal control. Negative control expression was set as 100% (***P < 0.001, *P < 0.05)

Many transcriptional target genes of the TGFβ signalling pathway have been reported to regulate angiogenesis.^30^, ^31^ Although predicted to be unlikely direct target genes of let‐7a, our interest remains in particular pro‐angiogenic candidate genes, such as *VEGFC*,* MMP9*,* ID1* and *PROX1*. We analysed their expression with qRT‐PCR after disruption of the let‐7a/TGFBR3 axis in HUVECs. All candidate target gene mRNA levels decreased in HUVECs transfected with let‐7a mimics (Figure 6A), suggesting that let‐7a indirectly inhibits expression of these four genes. Interestingly, in HUVECs transfected with siRNA targeting *TGFBR3*, only VEGFC and MMP9 mRNA levels significantly decreased, whereas mRNA levels of ID1 and PROX1 remained unchanged (Figure 6B). This observation indicates that TGFBR3 is required for optimal transcription of *VEGFC* and *MMP9* but not *ID1* and *PROX1*. Furthermore, we evaluated the protein levels of VEGFC and MMP9 in terms of mRNA levels showed that VEGFC and MMP9 are regulated by let‐7a/TGFBR3 axis (Figure 6C‐F). In summary, the data implicate that the let‐7a/TGFBR3 axis regulates angiogenesis through mediating transcriptional regulation of *VEGFC* and *MMP9*.

## 4 DISCUSSION

Dysregulated angiogenesis is considered to be one of the fundamental mechanisms regulating initiation and development of many diseases, such as cancer,^20^, ^32^, ^33^ heart disease^34^, ^35^ and inflammation.^36^, ^37^ In this study we found that *TGFBR3* is a novel target of let‐7a. Let‐7a suppresses tube formation and migration by directly targeting TGFBR3 in HUVECs, resulting in defective TGFβ signalling in vascular ECs. Therefore, we conclude that let‐7a likely suppresses angiogenesis via down‐regulation of *TGFBR3* expression, and the relative TGFβ signalling pathway may be implicated in its mechanism.

Involvement of miRNAs in angiogenesis was first studied in 2006. In past decade, researchers found early regulation of miRNAs might underpin the rescue of diabetes‐impaired angiogenesis.^38^ Poliseno et al. used miRNA microarray as a discovery tool and identified 27 highly expressed miRNAs in HUVECs; 15 of which were predicted to regulate expression of cell‐surface receptors of angiogenic factors.^17^ In pathological condition, the combination tamoxifen and/or letrozole with exercise training up‐regulated let‐7a expression, leading to reduced angiogenesis and tumour growth (microRNA‐206, let‐7a and microRNA‐21) pathways involved in the anti‐angiogenesis effects of the interval exercise training and hormone therapy in breast cancer.^19^ Let‐7 also repressed pathological ocular angiogenesis by targeting HMGA2. Overexpression of let‐7 by adenovirus suppressed endothelial cell migration and networking, blockade of let‐7a with anti‐miR promote migration.^39^ Let‐7a, one of the highly expressed miRNAs in HUVECs, has been identified to be an important inhibits role in multiple types. Let‐7a was shown to control Etv2 (Ets‐variant protein 2) expression and EC differentiation through a post‐transcriptional mechanism in zebrafish.^40^ Our in vivo and in vitro data were consistent with the previous study's conclusions that let‐7a imposed anti‐angiogenesis activity in a mouse model of breast cancer.^19^, ^41^ In particular, multiple evidence suggest let‐7a suppress cancer cell migration, invasion and proliferation. For instance, overexpression of let‐7a suppressed PTC cell migration and tumour growth by targeting AKT2.^42^ Exogenous IGF‐2 expression, a potent stimulus facilitated cancer progression, could reversed NEAT1‐knockdown‐induced growth inhibition, might be a direct target of let‐7a.^43^ Thus, overexpression of let‐7a suppressed cell migration and angiogenesis, which also supported our study that shown in Figure 2.

To explore the molecular mechanism of let‐7a in HUVECs, we used TargetScan and miRanda Web Servers, and reviewed considerable literature to screen its target genes in relation to angiogenesis. Several candidate genes were chosen for additional validation, which not only yielded high miRNA:mRNA alignment scores, but also belong to the TGFβ signalling pathway. After qRT‐PCR and Western blotting analyses, *TGFBR3* appeared to be a target gene of let‐7a owing to the suppression of its mRNA and protein levels by let‐7a overexpression. Furthermore, we identified the let‐7a‐binding site on the 3′UTR of *TGFBR3* and *Tgfbr3* mRNA and confirmed that it is indeed a direct target gene of let‐7a using reporter gene assay systems (Figure 1).

TGFBR3, also named betaglycan, is a type III transmembrane receptor of the TGFβ superfamily.^7^ TGFBR3 binds most TGFβ superfamily members with low affinity, but at high capacity and has demonstrated to act as a co‐receptor to augment signalling via typical TGFβ signalling after presenting ligands to the major TGFβ superfamily receptors.^44^, ^45^ A previous study showed that TβRIII can also undergo ectodomain shedding, which releases a soluble form that can bind TGF ligand in the extracellular domain, thereby reducing ligand availability to its receptors and inhibiting downstream signalling.^46^ Depletion of Tgfbr3 has led to mice embryonic lethal due to failed coronary vasculogenesis.^9^ Additional phenotypes associated with decreased proliferation and invasion of *Tgfbr3*‐knockout (KO) epicardial cells in vitro have been observed.^47^, ^48^ In zebrafish, knockdown of *Tgfbr3* by injection of antisense morpholinos lead to impaired angiogenesis in morphant embryos.^49^ Similarly, we knocked down the *TGFBR3* gene in HUVECs via transfecting siRNAs, resulting in decreased angiogenesis and migration (Figures 3 and 4). Particularly, we observed that let‐7a/TGFBR3 was required for hemangioendothelioma growth in the animal study, which could be an approach for in vivo strong evidence (Figure 5). Other studies utilizing gene KOs revealed that disrupting TGFβ signalling pathway components, including *Tgfbr2*,^50^ *Alk1* 51 and *Alk5* 52 (two types of *Tgfbr1*), and *Smad5*,^53^ results in defective vascular development. It was revealed that TGFBR3 directly down‐regulated by let‐7a might be essential for vascular tube formation via the TGFβ signalling pathway.

For further research, we selected several downstream target genes including VEGFC,^53^ MMP9,^54^ ID1^55^ and PROX1^56^ which have been demonstrated to be regulated by the TGFβ signalling pathway. It has been reported that ALK1 (one type of TGFBR1 in ECs) up‐regulates the expression of the pro‐angiogenic genes MMP9 and VEGFC, and siRNA‐mediated knockdown of MMP9 reduced VEGF‐induced angiogenesis in vitro.^53^, ^57^ TGFBR3, as a co‐receptor, could augment signalling via the typical TGFβ signalling pathway through SMADs activation after presenting ligand to TGFBR1 and TGFBR2. Based on the existing data, we speculated that let‐7a down‐regulates these genes by targeting TGFBR3 mRNA via the TGFβ signalling pathway. Through qRT‐PCR analysis, VEGFC and MMP9 were expectedly down‐regulated after transfection with let‐7a mimics or TGFBR3‐siRNA. However, reduction of ID1 and PROX1 mRNA levels were not significant. Furthermore, The Western blot showed the decreased expression of VEGFC and MMP9 (Figure 6), revealing the importance of MMP9 and VEGFC in angiogenesis. MMP9 and VEGF were down‐regulated by S100A4 silencing, resulting in suppression of cell proliferation, angiogenesis and invasion in thyroid cancer cells.^58^ Interestingly, preclinical data have reported the presence of a positive feedback regulation between VEGF and MMP9. MMP inhibitors were capable of inhibiting VEGF‐induced angiogenesis in vivo, indicating that MMP9 acts downstream of VEGF. Additionally, MMP9s in vivo angiogenic response was inhibited with a neutralizing VEGF antibody, suggesting that MMP9 also acts upstream of VEGF.^59^

In conclusion, our present study highlights that TGFBR3 is a novel and direct target gene of let‐7a and let‐7a suppresses endothelial angiogenesis and migration through the TGFβs signalling pathway. The TGFBR3/MMP9/VEGFC signalling pathway may be a potential therapeutic target for anti‐ or pro‐angiogenesis strategies in the survival and growth of solid cancers and other diseases. However, because we studied the mechanism of let‐7a on angiogenesis in vitro, further investigation is required in the future to determine whether it has a similar effect in vivo.

## CONFLICT OF INTEREST

The authors indicate no potential conflict of interest.

## AUTHOR CONTRIBUTION

S.W. and H.Z. conceived and directed the project; D.W., C.C., and Y.X. analysed the data; H.N. and Z.C. provided help with cloning; S.W., H.Z., K.D., X.C., and X.L. designed experiments, interpreted data and wrote the paper.

## References

[1] Risau W . Mechanisms of angiogenesis. Nature. 1997;386:671‐674. 9109485 10.1038/386671a0

[2] Carmeliet P , Jain RK . Angiogenesis in cancer and other diseases. Nature. 2000;407:249‐257. 11001068 10.1038/35025220

[3] Carmeliet P . Angiogenesis in health and disease. Nat Med. 2003;9:653‐660. 12778163 10.1038/nm0603-653

[4] Adams RH , Alitalo K . Molecular regulation of angiogenesis and lymphangiogenesis. Nat Rev Mol Cell Biol. 2007;8:464‐478. 17522591 10.1038/nrm2183

[5] Derynck R , Zhang YE . Smad‐dependent and Smad‐independent pathways in TGF‐beta family signalling. Nature. 2003;425:577‐584. 14534577 10.1038/nature02006

[6] Lopez‐Casillas F , Payne HM , Andres JL , Massague J . Betaglycan can act as a dual modulator of TGF‐beta access to signaling receptors: mapping of ligand binding and GAG attachment sites. J Cell Biol. 1994;124:557‐568. 8106553 10.1083/jcb.124.4.557 PMC2119924

[7] Bernabeu C , Lopez‐Novoa JM , Quintanilla M . The emerging role of TGF‐beta superfamily coreceptors in cancer. Biochem Biophys Acta. 2009;1792:954‐973. 19607914 10.1016/j.bbadis.2009.07.003

[8] Gatza CE , Oh SY , Blobe GC . Roles for the type III TGF‐beta receptor in human cancer. Cell Signal. 2010;22:1163‐1174. 20153821 10.1016/j.cellsig.2010.01.016 PMC2875339

[9] Compton LA , Potash DA , Brown CB , Barnett JV . Coronary vessel development is dependent on the type III transforming growth factor beta receptor. Circ Res. 2007;101:784‐791. 17704211 10.1161/CIRCRESAHA.107.152082

[10] Zamore PD , Haley B . Ribo‐gnome: the big world of small RNAs. Science. 2005;309:1519‐1524. 16141061 10.1126/science.1111444

[11] Mei J , Bachoo R , Zhang CL . MicroRNA‐146a inhibits glioma development by targeting Notch1. Mol Cell Biol. 2011;31:3584‐3592. 21730286 10.1128/MCB.05821-11 PMC3165557

[12] Reinhart BJ , Slack FJ , Basson M , et al. The 21‐nucleotide let‐7 RNA regulates developmental timing in *Caenorhabditis elegans* . Nature. 2000;403:901‐906. 10706289 10.1038/35002607

[13] Johnson CD , Esquela‐Kerscher A , Stefani G , et al. The let‐7 microRNA represses cell proliferation pathways in human cells. Cancer Res. 2007;67:7713‐7722. 17699775 10.1158/0008-5472.CAN-07-1083

[14] Song J , Cho KJ , Oh Y , Lee JE . Let7a involves in neural stem cell differentiation relating with TLX level. Biochem Biophys Res Comm. 2015;462:396‐401. 25976670 10.1016/j.bbrc.2015.05.004

[15] Tang R , Yang C , Ma X , et al. MiR‐let‐7a inhibits cell proliferation, migration, and invasion by down‐regulating PKM2 in gastric cancer. Oncotarget. 2016;7:5972‐5984. 26745603 10.18632/oncotarget.6821 PMC4868734

[16] Yang Q , Jie Z , Cao H , et al. Low‐level expression of let‐7a in gastric cancer and its involvement in tumorigenesis by targeting RAB40C. Carcinogenesis. 2011;32:713‐722. 21349817 10.1093/carcin/bgr035

[17] Poliseno L , Tuccoli A , Mariani L , et al. MicroRNAs modulate the angiogenic properties of HUVECs. Blood. 2006;108:3068‐3071. 16849646 10.1182/blood-2006-01-012369

[18] Brennan E , Wang B , McClelland A , et al. Protective effect of let‐7 miRNA family in regulating inflammation in diabetes‐associated atherosclerosis. Diabetes. 2017;66:2266‐2277. 28487436 10.2337/db16-1405

[19] Isanejad A , Alizadeh AM , Amani Shalamzari S , et al. MicroRNA‐206, let‐7a and microRNA‐21 pathways involved in the anti‐angiogenesis effects of the interval exercise training and hormone therapy in breast cancer. Life Sci. 2016;151:30‐40. 26924493 10.1016/j.lfs.2016.02.090

[20] Yan N , Wen L , Peng R , et al. Naringenin ameliorated kidney injury through let‐7a/TGFBR1 signaling in diabetic nephropathy. J Diabetes Res. 2016;2016:8738760. 27446963 10.1155/2016/8738760 PMC4944076

[21] Brennan EP , Nolan KA , Borgeson E , et al. ; GENIE Consortium . Lipoxins attenuate renal fibrosis by inducing let‐7c and suppressing TGFbetaR1. J Am Soc Nephrol. 2013;24:627‐637. 23520204 10.1681/ASN.2012060550 PMC3609134

[22] Wang B , Jha JC , Hagiwara S , et al. Transforming growth factor‐beta1‐mediated renal fibrosis is dependent on the regulation of transforming growth factor receptor 1 expression by let‐7b. Kidney Int. 2014;85:352‐361. 24088962 10.1038/ki.2013.372

[23] Lee SI , Jeon MH , Kim JS , Jeon IS , Byun SJ . The gga‐let‐7 family post‐transcriptionally regulates TGFBR1 and LIN28B during the differentiation process in early chick development. Mol Reprod Dev. 2015;82:967‐975. 26297836 10.1002/mrd.22575

[24] Wu T , Chen X , Peng R , et al. Let7a suppresses cell proliferation via the TGFbeta/SMAD signaling pathway in cervical cancer. Oncol Rep. 2016;36:3275‐3282. 27748903 10.3892/or.2016.5160

[25] Thompson CC , Ashcroft FJ , Patel S , et al. Pancreatic cancer cells overexpress gelsolin family‐capping proteins, which contribute to their cell motility. Gut. 2007;56:95‐106. 16847067 10.1136/gut.2005.083691 PMC1856675

[26] Gu R , Sun X , Chi Y , et al. Integrin beta3/Akt signaling contributes to platelet‐induced hemangioendothelioma growth. Sci Rep. 2017;7:6455. 28744026 10.1038/s41598-017-06927-0 PMC5527091

[27] Wu A , Wu K , Li J , et al. Let‐7a inhibits migration, invasion and epithelial‐mesenchymal transition by targeting HMGA2 in nasopharyngeal carcinoma. J Transl Med. 2015;13:105. 25884389 10.1186/s12967-015-0462-8 PMC4391148

[28] Hou X , Wan W , Wang J , et al. Let‐7a inhibits migration of melanoma cells via down‐regulation of HMGA2 expression. Am J Transl Res. 2016;8:3656‐3665. 27725848 PMC5040666

[29] Johnson SM , Grosshans H , Shingara J , et al. RAS is regulated by the let‐7 microRNA family. Cell. 2005;120:635‐647. 15766527 10.1016/j.cell.2005.01.014

[30] Ota T , Fujii M , Sugizaki T , et al. Targets of transcriptional regulation by two distinct type I receptors for transforming growth factor‐beta in human umbilical vein endothelial cells. J Cell Physiol. 2002;193:299‐318. 12384983 10.1002/jcp.10170

[31] Wu X , Ma J , Han JD , Wang N , Chen YG . Distinct regulation of gene expression in human endothelial cells by TGF‐beta and its receptors. Microvasc Res. 2006;71:12‐19. 16405919 10.1016/j.mvr.2005.11.004

[32] Powers JT , Tsanov KM , Pearson DS , et al. Multiple mechanisms disrupt the let‐7 microRNA family in neuroblastoma. Nature. 2016;535:246‐251. 27383785 10.1038/nature18632 PMC4947006

[33] Zheng Y , Li S , Ding Y , et al. The role of miR‐18a in gastric cancer angiogenesis. Hepatogastroenterology. 2013;60:1809‐1813. 24624454

[34] Nagpal V , Rai R , Place AT , et al. MiR‐125b is critical for fibroblast‐to‐myofibroblast transition and cardiac fibrosis. Circulation. 2016;133:291‐301. 26585673 10.1161/CIRCULATIONAHA.115.018174 PMC5446084

[35] Potus F , Ruffenach G , Dahou A , et al. Downregulation of microRNA‐126 contributes to the failing right ventricle in pulmonary arterial hypertension. Circulation. 2015;132:932‐943. 26162916 10.1161/CIRCULATIONAHA.115.016382

[36] Blaya D , Coll M , Rodrigo‐Torres D , et al. Integrative microRNA profiling in alcoholic hepatitis reveals a role for microRNA‐182 in liver injury and inflammation. Gut. 2016;65:1535‐1545. 27196584 10.1136/gutjnl-2015-311314

[37] Corsten M , Heggermont W , Papageorgiou AP , et al. The microRNA‐221/‐222 cluster balances the antiviral and inflammatory response in viral myocarditis. Eur Heart J. 2015;36:2909‐2919. 26206211 10.1093/eurheartj/ehv321

[38] Hourigan ST , Solly EL , Nankivell VA , et al. The regulation of miRNAs by reconstituted high‐density lipoproteins in diabetes‐impaired angiogenesis. Sci Rep. 2018;8:13596. 30206364 10.1038/s41598-018-32016-x PMC6133943

[39] Zhou Q , Frost RJA , Anderson C , et al. Let‐7 contributes to diabetic retinopathy but represses pathological ocular angiogenesis. Mol Cell Biol. 2017;37:e00001‐e00017. 10.1128/MCB.00001-17 PMC5533878 28584193

[40] Moore JC , Sheppard‐Tindell S , Shestopalov IA , Yamazoe S , Chen JK , Lawson ND . Post‐transcriptional mechanisms contribute to Etv2 repression during vascular development. Dev Biol. 2013;384:128‐140. 24036310 10.1016/j.ydbio.2013.08.028 PMC4139968

[41] Zhao W , Hu JX , Hao RM , et al. Induction of microRNAlet7a inhibits lung adenocarcinoma cell growth by regulating cyclin D1. Oncol Rep. 2018;40:1843‐1854. 30066899 10.3892/or.2018.6593 PMC6111629

[42] Zhou B , Shan H , Su Y , Xia K , Zou R , Shao Q . Let‐7a inhibits migration, invasion and tumor growth by targeting AKT2 in papillary thyroid carcinoma. Oncotarget. 2017;8:69746‐69755. 29050238 10.18632/oncotarget.19261 PMC5642513

[43] Qi L , Liu F , Zhang F , et al. lncRNA NEAT1 competes against let‐7a to contribute to non‐small cell lung cancer proliferation and metastasis. Biomed Pharmacother. 2018;103:1507‐1515. 29864936 10.1016/j.biopha.2018.04.053

[44] Kretzschmar M , Massague J . SMADs: mediators and regulators of TGF‐beta signaling. Curr Opin Genet Dev. 1998;8:103‐111. 9529613 10.1016/s0959-437x(98)80069-5

[45] Kirkbride KC , Townsend TA , Bruinsma MW , Barnett JV , Blobe GC . Bone morphogenetic proteins signal through the transforming growth factor‐beta type III receptor. J Biol Chem. 2008;283:7628‐7637. 18184661 10.1074/jbc.M704883200

[46] Elderbroom JL , Huang JJ , Gatza CE , et al. Ectodomain shedding of TbetaRIII is required for TbetaRIII‐mediated suppression of TGF‐beta signaling and breast cancer migration and invasion. Mol Biol Cell. 2014;25:2320‐2332. 24966170 10.1091/mbc.E13-09-0524 PMC4142606

[47] Sanchez NS , Hill CR , Love JD , et al. The cytoplasmic domain of TGF beta R3 through its interaction with the scaffolding protein, GIPC, directs epicardial cell behavior. Dev Biol. 2011;358:331‐343. 21871877 10.1016/j.ydbio.2011.08.008 PMC3183347

[48] Clark CR , Robinson JY , Sanchez NS , et al. Common pathways regulate type III TGFbeta receptor‐dependent cell invasion in epicardial and endocardial cells. Cell Signal. 2016;28:688‐698. 26970186 10.1016/j.cellsig.2016.03.004 PMC4827451

[49] Kamaid A , Molina‐Villa T , Mendoza V , et al. Betaglycan knock‐down causes embryonic angiogenesis defects in zebrafish. Genesis. 2015;53:583‐603. 26174808 10.1002/dvg.22876

[50] Oshima M , Oshima H , Taketo MM . TGF‐beta receptor type II deficiency results in defects of yolk sac hematopoiesis and vasculogenesis. Dev Biol. 1996;179:297‐302. 8873772 10.1006/dbio.1996.0259

[51] Urness LD , Sorensen LK , Li DY . Arteriovenous malformations in mice lacking activin receptor‐like kinase‐1. Nat Genet. 2000;26:328‐331. 11062473 10.1038/81634

[52] Larsson J , Goumans MJ , Sjostrand LJ , et al. Abnormal angiogenesis but intact hematopoietic potential in TGF‐beta type I receptor‐deficient mice. EMBO J. 2001;20:1663‐1673. 11285230 10.1093/emboj/20.7.1663 PMC145465

[53] Niessen K , Zhang G , Ridgway JB , Chen H , Yan M . ALK1 signaling regulates early postnatal lymphatic vessel development. Blood. 2010;115:1654‐1661. 19903896 10.1182/blood-2009-07-235655 PMC2830767

[54] Xu J , Zhu D , Sonoda S , et al. Over‐expression of BMP4 inhibits experimental choroidal neovascularization by modulating VEGF and MMP‐9. Angiogenesis. 2012;15:213‐227. 22392094 10.1007/s10456-012-9254-4 PMC3413482

[55] Xiao F , Qiu H , Cui H , et al. MicroRNA‐885‐3p inhibits the growth of HT‐29 colon cancer cell xenografts by disrupting angiogenesis via targeting BMPR1A and blocking BMP/Smad/Id1 signaling. Oncogene. 2015;34:1968‐1978. 24882581 10.1038/onc.2014.134

[56] Yoshimatsu Y , Lee YG , Akatsu Y , et al. Bone morphogenetic protein‐9 inhibits lymphatic vessel formation via activin receptor‐like kinase 1 during development and cancer progression. Proc Natl Acad Sci USA. 2013;110:18940‐18945. 24133138 10.1073/pnas.1310479110 PMC3839734

[57] Gupta A , Zhou CQ , Chellaiah MA . Osteopontin and MMP9: associations with VEGF expression/secretion and angiogenesis in PC3 prostate cancer cells. Cancers. 2013;5:617‐638. 24216994 10.3390/cancers5020617 PMC3730333

[58] Jia W , Gao XJ , Zhang ZD , Yang ZX , Zhang G . S100A4 silencing suppresses proliferation, angiogenesis and invasion of thyroid cancer cells through downregulation of MMP‐9 and VEGF. Eur Rev Med Pharmacol Sci. 2013;17:1495‐1508. 23771538

[59] Ebrahem Q , Chaurasia SS , Vasanji A , et al. Cross‐talk between vascular endothelial growth factor and matrix metalloproteinases in the induction of neovascularization in vivo. Am J Pathol. 2010;176:496‐503. 19948826 10.2353/ajpath.2010.080642 PMC2797907

